# Atypical Erysipelas With Serohematic Bullae and Necrosis: A Delayed Diagnostic and Therapeutic Challenge

**DOI:** 10.7759/cureus.91362

**Published:** 2025-08-31

**Authors:** Brigida Tueros, Alejandra Vargas, Fiorella Rodriguez, Irene Fernández Ponce

**Affiliations:** 1 General Medicine, Universidad Nacional Federico Villarreal, Lima, PER; 2 General Medicine, San Juan Bautista Private University, Lima, PER; 3 Medical School, Universidad de Aquino Bolivia, Santa Cruz de la Sierra, BOL; 4 Obstetrics and Gynecology, Hospital San Isidro Labrador, Lima, PER

**Keywords:** atypical skin infection, bullous erysipelas, delayed diagnosis, lower limb cellulitis differential, post-infectious neuropathic pain, streptococcus pyogenes infection

## Abstract

Erysipelas is a superficial bacterial skin infection, most frequently caused by *Streptococcus pyogenes*, and typically presents with well-demarcated erythema, commonly affecting the lower extremities. Although usually responsive to early antibiotic therapy, atypical variants such as bullous erysipelas may complicate the clinical picture and delay appropriate management. We present the case of a 60-year-old woman with underlying comorbidities who developed a progressively worsening lower limb infection following minor trauma. The initial misdiagnosis and suboptimal antibiotic therapy contributed to clinical deterioration, including the formation of bullae and necrotic areas, raising suspicion for more severe infections. Despite negative cultures and biopsy results, clinical features supported the diagnosis of bullous erysipelas, which responded favorably to intravenous and oral β-lactam antibiotics. Long-term follow-up revealed persistent neuropathic pain, highlighting the potential for chronic sequelae even after resolution of the infection. This case emphasizes the need for early recognition of atypical erysipelas, timely initiation of appropriate antibiotics, and ongoing multidisciplinary management to address complications and optimize patient outcomes.

## Introduction

Skin and soft tissue bacterial infections represent a broad group of microbial diseases in which pathogens spread through various anatomical layers of the skin and soft tissues and may even disseminate systemically. Common entry points include disruptions in the skin barrier, such as interdigital fungal infections, minor trauma, wounds, insect bites, or injections [1,2]. One such condition is erysipelas, a skin infection that primarily involves the dermis and the superficial cutaneous lymphatic vessels. It is mainly caused by *Streptococcus pyogenes*, a group A β-hemolytic streptococcus [3,4].

Clinically, erysipelas is characterized by a well-demarcated, elevated area of erythema, most frequently affecting the lower limbs. Diagnosis is primarily clinical and may occur at any age, though it is more common in children under three years old and in adults around 60 years of age [3,5]. Epidemiologic data suggest variability by sex: some studies report a female predominance, especially in older adults, whereas others describe higher rates in younger males, likely reflecting differences in study populations and settings [5,6].

Erysipelas presents a diagnostic challenge as it is often confused with cellulitis. Both are acute bacterial skin infections, but erysipelas typically presents with raised, sharply demarcated borders, fever, and a more rapid onset, whereas cellulitis involves the deeper dermis and subcutaneous tissue, showing less distinct margins and slower progression [2,7].

A less common but clinically significant variant is bullous erysipelas, estimated to occur in approximately 5-10% of erysipelas cases [8]. The proposed pathophysiology involves toxin-mediated epidermal necrosis and increased vascular permeability leading to the accumulation of serous or serohematic fluid within bullae [8]. Reported risk factors include advanced age, diabetes mellitus, immunosuppression, and delayed initiation of antibiotic therapy [8]. This presentation is considered atypical and may complicate the clinical picture, delaying diagnosis and treatment.

While erysipelas can be severe, it is rarely fatal and typically responds quickly and favorably to antibiotic therapy [4,7].

In this report, we present the case of a 60-year-old female who, following minor trauma to her left leg, developed a delayed diagnosis of bullous erysipelas with atypical serohematic bullae. This case highlights the diagnostic difficulties associated with this rare form and underscores the importance of early recognition and appropriate management to prevent potentially life-threatening complications.

## Case presentation

**Figure 1:**
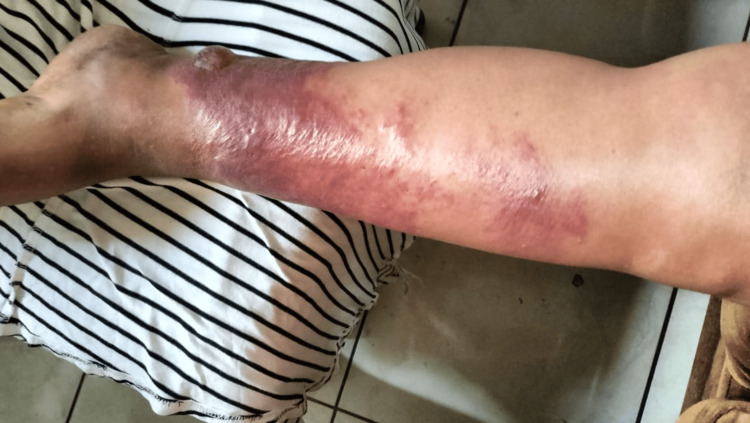
Initial Presentation of Bullous Erysipelas Early-stage erysipelas presents as a large, well-demarcated, erythematous, and edematous plaque on the left lower leg, characterized by purplish discoloration and shiny, stretched skin. The lesion appeared shortly after minor trauma and progressively worsened despite initial antibiotic treatment, eventually evolving into bullous and necrotic forms.

A 60-year-old woman with a medical history of fibromyalgia, hypertension, and grade I obesity sustained trauma to her left leg after being struck by a rusty metal sheet. She developed pain, a burning sensation, and erythema at the site of injury. She presented to the trauma department, where a left leg X-ray showed no evidence of fracture. She was prescribed clindamycin 300 mg every eight hours for 10 days and naproxen 500 mg every 12 hours for five days. However, she did not complete this treatment because her symptoms rapidly worsened. The following day, she was evaluated by dermatology, where a clinical diagnosis of cellulitis was made, and she was prescribed amoxicillin/clavulanic acid 500 mg every 12 hours for five days. She only received one dose, as that same evening her clinical condition deteriorated, with the development of extensive erythema and violaceous discoloration of the leg (Figure 1).

**Figure 2:**
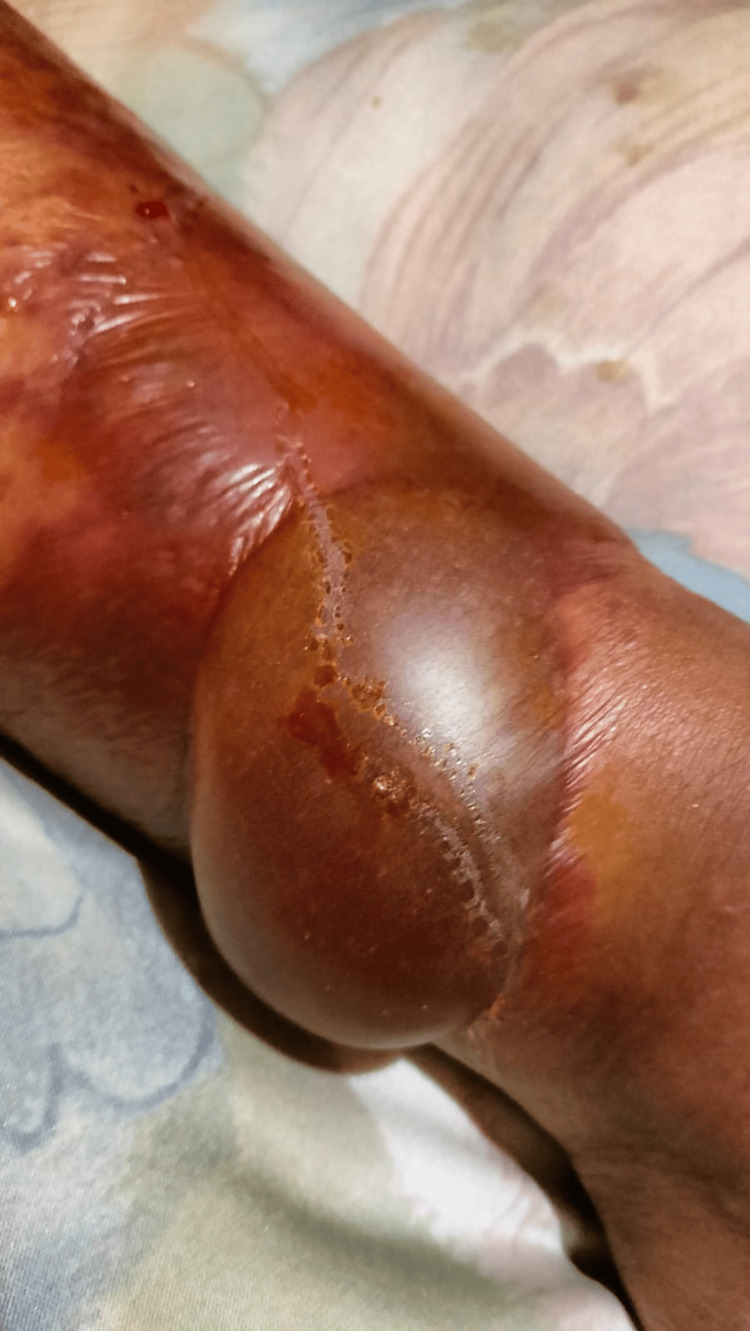
Hemorrhagic Bullous Lesion in Advanced Bullous Erysipelas A large, tense, hemorrhagic bulla located on the anterior aspect of the left lower leg, surrounded by extensive erythema, violaceous discoloration, and areas of skin necrosis. The lesion developed progressively following inadequate initial antibiotic treatment and was initially mistaken for necrotizing fasciitis before a final diagnosis of bullous erysipelas was made.

She was admitted the next day due to rapid progression of the lesion, which now presented with necrotic areas, bullae, and a 5 cm serohematic mass on the medial aspect of the left ankle (Figure 2).

Laboratory evaluation, including complete blood count and glucose levels, was within normal limits. Wound cultures and a skin biopsy were performed but showed no significant findings. At this point, intravenous ceftriaxone (2 g/day) was initiated and continued for 10 days.

**Figure 3:**
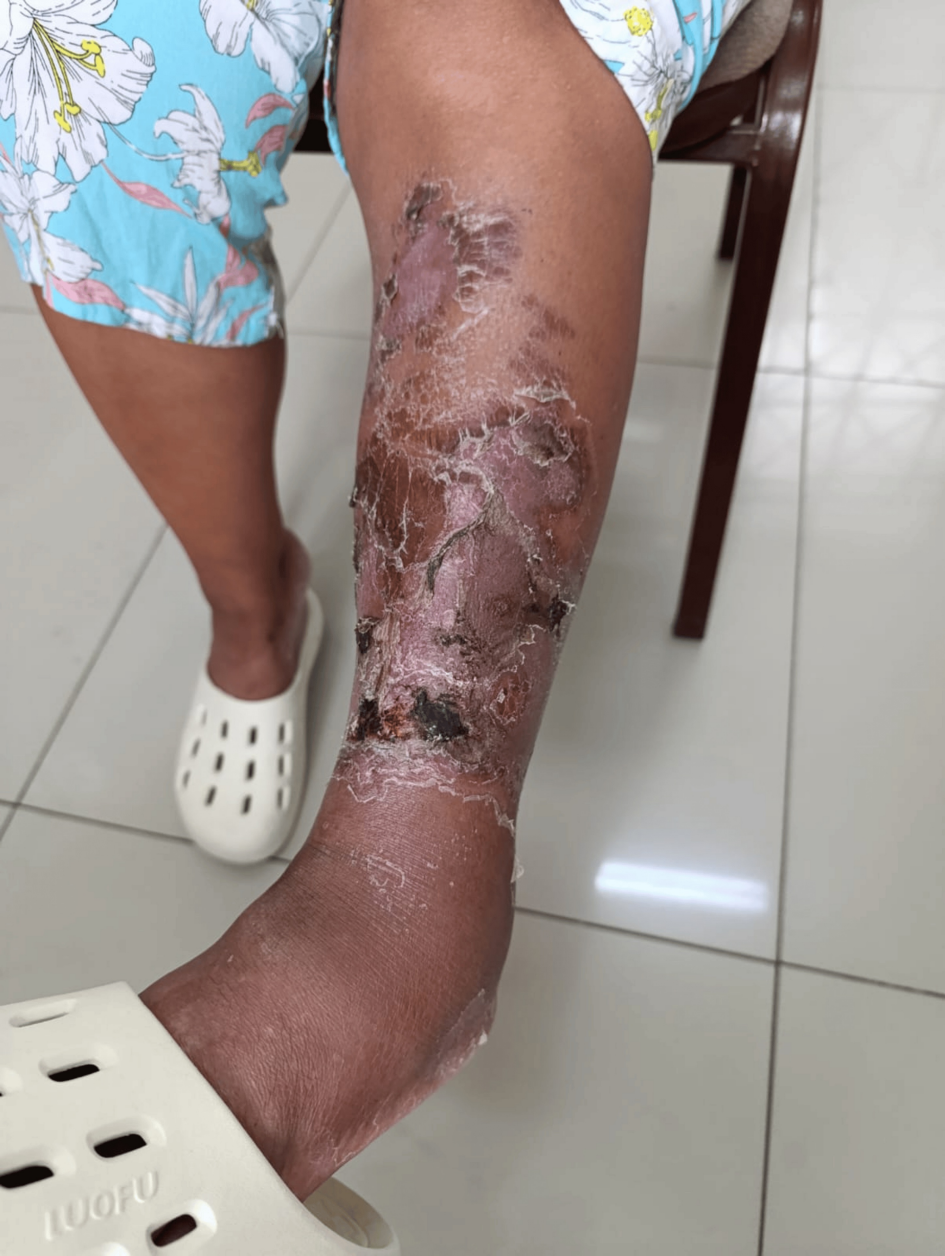
Healing Phase of Bullous Erysipelas After Bulla Rupture Post-inflammatory changes in the left lower leg, showing desquamation, crusted erosions, hyperpigmentation, and areas of residual necrosis. This image represents the late stage of bullous erysipelas after spontaneous rupture of hemorrhagic bullae, with signs of re-epithelialization and scabbing during the healing process.

During hospitalization, dermatology and infectious disease consultations were obtained. Initially, necrotizing fasciitis was considered due to the rapid evolution and necrosis, particularly during the last five days of hospitalization. However, the final diagnosis by the infectious disease team was bullous erysipelas. Following clinical improvement, the patient was discharged with oral cephalexin 500 mg every six hours for nine days. At outpatient follow-up, the patient showed resolution of the acute infection, with the affected area in the recovery phase characterized by desquamation and post-inflammatory changes (Figure 3).

Nevertheless, she continued to experience burning pain and decreased sensitivity in the affected region, consistent with post-infectious neuropathic symptoms. These were managed with gabapentin, with partial but progressive improvement.

## Discussion

Erysipelas is a common skin infection that involves the superficial dermis [9]. In general, it responds well to antibiotic treatment; however, if left untreated, mortality can reach up to 11% [10]. Clinically, it is characterized by an erythematous lesion with well-demarcated borders [11] and is considered a "mild" form of cellulitis, since deeper tissue involvement is absent [12].

Predisposing factors are found in up to 78% of patients. The lower extremities are most commonly affected (66%), and approximately 62% of cases yield a positive culture. The most frequently isolated pathogens include *Streptococcus pyogenes *(GAS), *Staphylococcus aureus*, and group B streptococcus (GBS). Less commonly, group C streptococcus (GCS), *Pseudomonas aeruginosa*, and* Enterococcus *spp. have been reported [13].

Diagnosis is primarily clinical, based on the presence of an erythematous lesion with well-defined borders in a patient with risk factors. However, laboratory criteria have also been proposed to support the diagnosis [14]. Differential diagnoses include herpes zoster, acute dermatitis, acute gout, septic arthritis, deep vein thrombosis, and cellulitis (the latter typically showing ill-defined borders and involvement of deeper dermis) [15].

In this case, the patient had a history of cutaneous trauma and subsequently developed an erythematous lesion with well-defined borders and bullae with serohematic content. He was hospitalized and treated empirically with intravenous ceftriaxone for 12 days, with favorable evolution. The average treatment duration reported in the literature is approximately 11 days [16]. Two cultures of the lesion were performed, both of which were negative, most likely because the patient had already received antibiotics before sampling.

Bullous erysipelas is uncommon and may cause diagnostic confusion. A detailed history and comprehensive physical examination are essential to establish the correct diagnosis.

The patient initially received amoxicillin/clavulanic acid, a broad-spectrum antibiotic with activity against* S. pyogenes* and *S. aureus*, the most frequent pathogens in erysipelas. Treatment failure can be explained by several factors: (1) Initial misdiagnosis: the patient was initially classified as having cellulitis, which influenced the therapeutic choice. Since the bacterial cell wall is crucial for* S. pyogenes* virulence, clindamycin, acting on protein synthesis rather than the cell wall, is not as bactericidal as β-lactams; (2) delayed initiation of appropriate therapy: although amoxicillin/clavulanic acid, penicillin, and first-generation cephalosporins [17] provide good coverage, late administration may have contributed to complications. Prior use of clindamycin and discontinuation of amoxicillin/clavulanic acid likely reduced the bacterial load without eradicating the infection, allowing for atypical progression; and (3) a high initial bacterial load trauma with a rusty metal sheet may have facilitated colonization by more aggressive or resistant bacteria.

The condition progressed with serohematic bullae and necrotic areas, initially raising suspicion of necrotizing fasciitis. Despite negative cultures, the rapid progression and severity of lesions justified hospitalization and intravenous ceftriaxone administration for 10 days.

Early recognition of warning signs, such as rapid lesion progression, necrosis, and a lack of response to oral antibiotics, could have shortened hospitalization. Close monitoring and earlier escalation to intravenous antibiotics might have prevented clinical deterioration. Furthermore, risk factors for complications have been identified, including age ≤50 years, female sex, heart disease, smoking, and prior antibiotic or NSAID (nonsteroidal anti-inflammatory drug) use before hospitalization, several of which were present in this patient.

Cases of hemorrhagic bullous erysipelas have been reported in kidney transplant recipients, and other case series highlight the low incidence but clinical relevance of this variant [17]. Nevertheless, there is still insufficient evidence to determine whether bullous erysipelas implies a more severe course or requires a different therapeutic approach.

This case emphasizes the importance of considering atypical variants of erysipelas in patients with risk factors such as obesity, previous trauma, and poor response to oral antibiotics. Early recognition and timely initiation of targeted therapy are critical to reducing complications and avoiding prolonged hospitalization.

## Conclusions

This case highlights the importance of early recognition and appropriate antibiotic selection in erysipelas. While typically manageable on an outpatient basis, the bullous variant accounts for 5-15% of cases and may progress to severe forms with necrosis. In our patient, the late development of serohematic bullae and necrosis led to consideration of necrotizing fasciitis only during the final days of hospitalization, underscoring the diagnostic challenges in atypical presentations. Although the absence of microbiological confirmation is a limitation, the favorable response to intravenous cephalosporins supports a streptococcal etiology. This report contributes to the limited published descriptions of complicated bullous erysipelas, emphasizing the need for heightened clinical suspicion and more precise protocols to improve outcomes in unusual cases.
